# Utility of the ISTH bleeding assessment tool in predicting platelet defects in participants with suspected inherited platelet function disorders

**DOI:** 10.1111/jth.12332

**Published:** 2013-09-12

**Authors:** G C Lowe, M Lordkipanidzé, S P Watson on behalf of the uk gapp study group

**Affiliations:** Centre for Cardiovascular Sciences, Institute of Biomedical Research, College of Medical and Dental Sciences, University of BirminghamEdgbaston, Birmingham, UK

**Keywords:** Blood platelet disorders, Coagulation disorders, inherited, Decision support techniques, Platelet aggregation, Platelet function tests

## Abstract

**Background:**

The ISTH bleeding assessment tool (ISTH-BAT) was developed to record bleeding symptoms and to aid diagnosis in patients with a possible bleeding disorder.

**Objectives:**

To investigate the utility of the ISTH-BAT in predicting functional defects in platelet activation in participants with suspected inherited platelet function disorders.

**Patients/Methods:**

Participants with clinical evidence of excessive bleeding and suspected inherited platelet function disorders and healthy volunteers were recruited to the Genotyping and Phenotyping of Platelets study (GAPP; ISRCTN 77951167). The ISTH-BAT questionnaire was applied by a trained investigator prior to lumiaggregometry.

**Results:**

One hundred participants were included (79 with suspected inherited platelet function disorders, and 21 healthy volunteers). The ISTH-BAT score in participants with suspected inherited platelet function disorders (median 12; interquartile range [IQR] 8–16) was significantly higher than in healthy volunteers (median 0; IQR 0–0). There was no difference between participants with suspected inherited platelet function disorders with a platelet defect detected by lumiaggregometry (median 11; IQR 8–16) and those with normal platelet function (median 12; IQR 8–14) (*P* > 0.05). The ISTH-BAT score was not associated with a demonstrable platelet defect on platelet function testing (area under the receiver operating characteristic curve = 0.501 [95% confidence interval 0.372–0.630, *P* = 0.98] and odds ratio 1.01 [95% confidence interval 0.93–1.09, *P* = 0.91]).

**Conclusions:**

The ISTH-BAT is a powerful tool for documenting lifelong bleeding history. However, the score obtained is not predictive of the presence of a platelet defect on lumiaggregometry in patients with suspected inherited platelet function disorders.

## Introduction

Recent years have seen the development of many forms of bleeding assessment tool (BAT) with the aim of standardizing the documentation of symptoms of excessive bleeding in patients, allowing comparisons between patients and the selection of patients in whom further investigations are appropriate. The evolution and validation of these tools, along with the rapid progress in the field, was covered in a recent comprehensive review 1. Many of the tools developed have been specific to certain bleeding disorders, such as von Willebrand disease 2,3, or have been targeted at certain populations, such as children 4–6 or women with menorrhagia 7,8. International collaboration has been recognized as being essential in the further development of these tools, owing to the rarity of some inherited bleeding disorders.

The ISTH-BAT was developed as a tool with which to accurately record bleeding symptoms in all hemorrhagic disorders and to aid diagnosis in patients referred with a possible bleeding disorder 9. It was designed to capture the importance of recurrent minor bleeds in addition to more severe hemorrhage, including life-threatening episodes. It is also a potential research tool in studies that examine patients with excessive bleeding, allowing documentation of the severity of bleeding and comparison between patient groups across and within studies. A recent study has shown the ISTH-BAT to be helpful in assessing children and parents in routine clinical practice, and has found it to be equally discriminative as a BAT specifically designed for pediatric patients with regard to the prevalence of an underlying bleeding disorder 10. However, it has not yet been validated in a large cohort of patients with suspected inherited platelet function disorders.

## Materials and methods

### Participant selection

Participants with suspected inherited platelet function disorders were recruited to the Genotyping and Phenotyping of Platelets study (GAPP; ISRCTN 77951167) from October 2011 to December 2012 from UK Comprehensive Care Haemophilia Centres, and were invited to participate in this study if they satisfied the following criteria:

1. Abnormal bleeding symptoms compatible with a possible inherited platelet function disorder (spontaneous mucocutaneous bleeding or abnormal bleeding at other sites following trauma or invasive procedures). Patients with existing diagnoses of Glanzmann's thrombasthenia, Bernard–Soulier syndrome, May–Hegglin anomaly or Hermansky–Pudlak syndrome were excluded.
2. Platelet count within the local laboratory reference range.
3. Results from coagulation factor tests within local laboratory reference intervals (minimum panel of prothrombin time, activated thromboplastin time, Clauss fibrinogen activity, von Willebrand factor antigen level, ristocetin cofactor activity, and factor VIII:C activity)

Laboratory testing was deferred in participants exposed within 2 weeks to drugs known to affect platelet function.

Healthy volunteers aged ≥ 18 years were included in this study. Participants were considered to be healthy if they had not previously required clinical consultation or investigation for excessive bleeding, did not require long-term medical therapy, and had refrained from using drugs known to influence platelet function in the previous 2 weeks.

This study was approved by the National Research Ethics Service Committee West Midlands – Edgbaston (REC reference: 06/MRE07/36), and all participants gave written informed consent in accordance with the Declaration of Helsinki.

### Assessment of bleeding history

The ISTH-BAT was used for assessment of the participants' bleeding history. The questionnaire was applied for patients with suspected inherited platelet function disorders (from UK Comprehensive Care Haemophilia Centres) and for healthy volunteers by trained personnel who regularly took bleeding histories for study participants. The bleeding assessment scores were compiled prior to platelet function testing, so that investigators were not influenced by lumiaggregometry results when assessing and quantifying the bleeding history. The ISTH-BAT score can range from 0 to 56 in women and from 0 to 48 in men, based on a maximal score of 4 in 14 separate categories assessing different types of bleeding.

### Assessment of platelet function

Blood was drawn by venepuncture through a 21-gauge needle into evacuated tubes containing 3.8% sodium citrate. Platelet function was assessed with light transmission aggregometry as described previously 11. Platelet-rich plasma was prepared by centrifugation of whole blood at 200 ×*g* for 20 min, and autologous platelet-poor plasma was obtained by further centrifugation at 1000 ×*g* for 10 min. A dual-channel Chronolog lumiaggregometer (Model 460 VS; Chronolog, Havertown, PA, USA) was used to measure platelet aggregation in response to: 3, 10 and 30 μm ADP; 10 and 30 μm adrenaline; 0.5 and 1 mm arachidonic acid; 1 and 3 μg/mL collagen; 30 and 100 μm protease-activated receptor 1-specific peptide (SFLLRN); and 1.5 mg/mL ristocetin. ATP secretion was assessed with the Luciferin–Luciferase reagent (Chronolume, Chrono-log, Havertown, PA, USA) and an ATP standard solution. Results were classified as abnormal by reference to a bank of local healthy volunteers 11. Platelet function testing operators were kept blind of the ISTH-BAT score.

### Statistical analyses

Normally distributed continuous variables are presented as mean ± standard deviation, non-normally distributed continuous variables as median (interquartile range [IQR]), and categorical variables as frequencies (percentages). Variables were analyzed for a normal distribution with the Kolmogorov–Smirnov test. The association between the presence of platelet defects as assessed by platelet function testing and the ISTH-BAT score was determined with the non-parametric Kruskall–Wallis test, with Dunn's adjustment for multiple comparisons. To evaluate the ISTH-BAT's ability to discriminate between patients with and without a demonstrable platelet defect on platelet function testing, a receiver operating characteristic (ROC) curve analysis was performed, and a simple logistic regression was used. A two-sided *P*-value of < 0.05 was considered to be significant. Analyses were performed with spss 14.0 for Windows (SPSS Institute, Chicago, IL, USA) and graphpad prism 5 for Windows (GraphPad Software, San Diego, CA, USA).

## Results

### Participant characteristics

Of the 100 participants studied, 21 were healthy volunteers and 79 were recruited from UK Comprehensive Care Haemophilia Centres with suspected inherited platelet function disorders. The median age of participants with bleeding symptoms was 39 years (IQR 31–53 years), and that of healthy volunteers was 32 years (IQR 30–35 years). The majority of recruited participants were female (81% of participants with bleeding symptoms, and 67% of healthy volunteers).

### Association of scores with platelet function testing results

**Figure 1:**
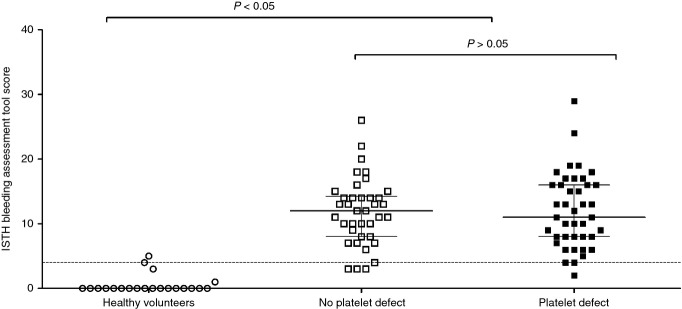
Association between the presence of a platelet function defect on lumiaggregometry and the ISTH bleeding assessment tool score. 95th percentile (score of 4) calculated from healthy volunteers and represented by the horizontal dotted line. For the data points, the line represents the median and the whiskers represent the interquartile range. Statistical analysis was performed with the non-parametric Kruskall–Wallis test, with Dunn's adjustment for multiple comparisons.

**Figure 2:**
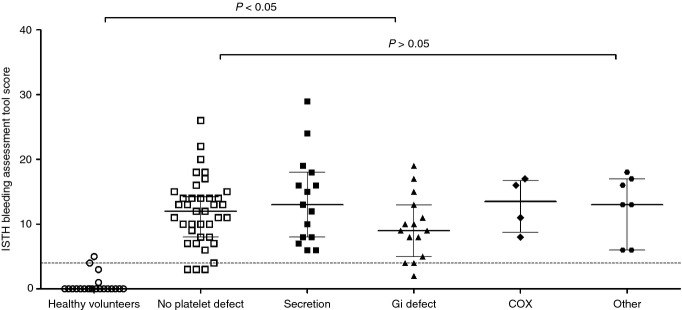
Association between type of platelet function defect on lumiaggregometry and the ISTH bleeding assessment tool score. 95th percentile (score of 4) calculated from healthy volunteers and represented by the horizontal dotted line. For the data points, the line represents the median and the whiskers represent the interquartile range. Statistical analysis was performed with the non-parametric Kruskall–Wallis test, with Dunn's adjustment for multiple comparisons. For a description of the types of defect, see 11. COX, cyclooxygenase; Gi, abnormality in Gi signalling (predominantly seen as changes in aggregation to ADP and adrenaline).

A platelet defect was found on lumiaggregometry in 52% of participants with bleeding symptoms, in accordance with our previously published findings 11. The score obtained with the ISTH-BAT in participants referred by UK Comprehensive Care Haemophilia Centres with bleeding symptoms (12; IQR 8–16) was significantly higher than in healthy volunteers (0; IQR 0–0), consistent with the clinical diagnosis of a bleeding disorder. However, there was no significant difference between participants in whom a platelet defect was detected by lumiaggregometry (11; IQR 8–16) and those in whom platelet function was deemed to be normal (12; IQR 8–14) (Fig. 1). There was no association between the type of platelet defect detected by lumiaggregometry (as defined in our recent study 11) and the ISTH-BAT score, although the numbers of patients in some diagnostic classification subgroups was relatively small (Fig. 2).

**Figure 3:**
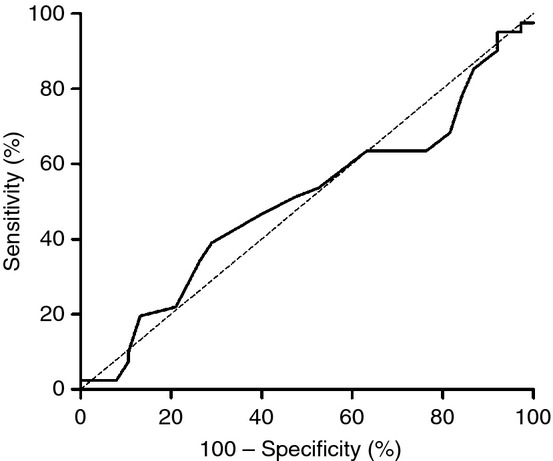
Receiver operating characteristic curve for the presence of a platelet function defect on lumiaggregometry and the ISTH bleeding assessment tool score. Area under the curve: 0.50 (95% confidence interval 0.372–0.63), *P* = 0.98.

A ROC curve analysis was carried out to evaluate whether the ISTH-BAT could discriminate between patients with and without a demonstrable platelet defect on lumiaggregometry (Fig. 3). The area under the curve was 0.50 (95% confidence interval [CI] 0.37–0.63, *P* = 0.98), demonstrating a lack of discriminative ability. Moreover, the ISTH-BAT score was not associated with a demonstrable platelet defect on platelet function testing in a simple logistic regression model (odds ratio 1.01, 95% CI 0.93–1.09, *P* = 0.91).

Subgroup analyses were performed to examine whether certain bleeding symptoms were predictive of the presence of a platelet defect on lumiaggregometry. ISTH-BAT scores for epistaxis, cutaneous bleeding, postoperative (including post-dental work) bleeding and menorrhagia in women were assessed (Table 1). No statistical significance was found for any of these subgroups.

**Table 1:** Subgroup analyses for the ISTH bleeding assessment tool score in patients with and without a platelet defect

Bleeding symptom	Patients with platelet defects (median, interquartile range)	Patients without platelet defects (median, interquartile range)	*P*-value
Epistaxis	1, 0–3	1, 0–2	0.67
Cutaneous bleeding	1, 0–2	1, 0–1	0.17
Postoperative bleeding (including post-dental work)	4, 2–6	4, 2–6	0.89
Menorrhagia	3, 1–4	3, 2–4	0.27

Symptoms assessed were epistaxis (maximum score of 4), cutaneous bleeding (maximum score of 4), postoperative bleeding (including post-dental work, maximum score of 8), and menorrhagia (in female participants only, maximal score of 4). Statistical analyses were performed with the non-parametric Mann–Whitney *U*-test.

The sensitivity, specificity, positive predictive value and negative predictive value of the ISTH-BAT in predicting the presence of a platelet defect on lumiaggregometry was assessed (Table 2). A cut-off value of 12 was used for the ISTH-BAT score, as this represented the 50th centile of ISTH-BAT score in all patients. This analysis confirmed the absence of association between a high ISTH-BAT score and the likelihood of having a platelet defect (*κ* = − 0.04, 95% CI − 0.26 to 0.18).

**Table 2:** The predictive value of an ISTH bleeding assessment tool (ISTH-BAT) score of ≥ 12 (2 × 2 analysis) in predicting the presence of a platelet defect on lumiaggregometry

	Defect present on lumiaggregometry	Defect absent on lumiaggregometry	
ISTH-BAT score of ≥ 12	20	20	Positive predictive value = 50%
ISTH-BAT score of < 12	21	18	Negative predictive value = 54%
	Sensitivity = 49%	Specificity = 53%	

An ISTH-BAT score of 12 represents the 50th centile in all patients studied. Kappa statistic for concordance = − 0.04 (95% confidence interval − 0.26 to 0.18), demonstrating a lack of significance.

## Discussion

Our findings confirm that the ISTH-BAT score is raised in patients with clinical evidence of excessive bleeding and a suspected platelet function disorder. We have also shown that healthy volunteers have a low score (≤ 4), in keeping with the absence of a history of bleeding episodes. However, the score is unable to discriminate between patients with clinically diagnosed bleeding disorders subsequently shown to have a platelet defect on lumiaggregometry and those in whom an underlying platelet defect was not detected. This suggests that the ISTH-BAT is powerful in documenting bleeding symptoms, but that its level as such is not predictive of the likelihood of diagnosing an inherited platelet disorder in patients with excessive bleeding. It is possible that some patients classified as not having a platelet defect do have a mild defect that is below the limit of sensitivity of our current testing assays, or have a defect in other aspects of platelet function (such as procoagulant activity) that was not tested for. Given the history of excessive bleeding in all patients, abnormalities in other untested parts of the hemostatic pathway may account for the clinical presentation of a proportion of the patients in whom we did not detect a platelet defect.

The healthy volunteer group included in this study is small (*n* = 21). The 95th centile for the ISTH-BAT score in this group is 4, although defining a more precise upper limit of normal range for the ISTH-BAT score would require larger studies that would ideally examine heterogeneous populations. In addition, the median age in the healthy volunteer group was slightly lower than in the patient group. This may have reduced the ISTH-BAT score in healthy volunteers, owing to fewer cumulative challenges to the hemostatic system, such as surgery or pregnancy and childbirth in women. Previous work has shown that platelet aggregability slightly increases with age 12, meaning that the aggregation ranges observed in the healthy volunteer group may be lower than would be expected in the patients if platelet function was normal. However, this would have biased the results in the patient group towards increased platelet reactivity, and therefore should not have led to mislabeling of patients with normal responses as having a platelet defect.

The documentation of bleeding risk in mild bleeding disorders is challenging. Mucocutaneous bleeding symptoms are reported in a significant proportion of the healthy population, which makes determination of the threshold that constitutes excessive bleeding difficult 13. Mild bleeding disorders are prevalent in the general population, reaching up to 1%, but both the clinical diagnosis and laboratory characterization remain complicated 14. The presence of a laboratory phenotype does not necessarily lead to bleeding, especially in the absence of a hemostatic challenge, and, as a consequence, most mild bleeding disorders, particularly inherited platelet disorders, remain clinically and biochemically ill-defined 14,15. There is an important clinical need for an assessment tool that would allow clinical characterization of this population, in order to stratify which patients require further investigation.

Numerous scores for bleeding assessment have been proposed over the years (reviewed in 15 and 1). The strength of the ISTH-BAT is in specifically capturing the frequency of mild bleeding symptoms, which is often missed by traditional BATs 9. As such, the ISTH-BAT is particularly relevant to patients with recurrent and mild bleeding symptoms, which constitute a key feature of inherited platelet disorders 14,15.

There is little prior experience with this tool in mild bleeding disorders. Using a previous version of a BAT based on the European MCMDM-1 VWD study 3, Tosetto *et al*. 16 analyzed the BAT's clinical utility in 215 patients with mild bleeding disorders. The findings supported the use of the BAT, as a low bleeding score could reasonably exclude the presence of a mild bleeding disorder in an unselected population, and the utility increased when the BAT score was used in conjunction with laboratory testing 16. Our results agree with these findings, as patients with clinical evidence of excessive bleeding in our study did have significantly higher ISTH-BAT scores than healthy volunteers. A further study by Bidlingmaier *et al*. 10 looked at the clinical utility of the ISTH-BAT questionnaire in a routine pediatric setting. In this study on 100 children presenting with a mild bleeding diathesis, a low ISTH-BAT score made a bleeding disorder diagnosis unlikely, consistent with our own findings and those of Tosetto *et al*. 10,16. However, only five children were diagnosed with a possible platelet disorder in this study, thus reducing the generalizability to a large cohort of patients with suspected inherited platelet function disorders. Also, given that the ISTH-BAT assesses responses to hemostatic challenges, including menorrhagia, childbirth, dental procedures, and surgery, the applicability of the scores obtained in children to an adult population is uncertain, as many children will not yet have been exposed to these challenges.

Our study is the first to report on the utility of the ISTH-BAT in a large cohort of patients with suspected inherited platelet function disorders. Our findings support the use of the ISTH-BAT to document recurrent bleeding symptoms in this population. However, the ISTH-BAT score is not predictive of which patients have a platelet function defect determined by laboratory investigation with lumiaggregometry, which is the currently accepted gold standard for assessing platelet function.

## Conclusion

Assessment of symptoms with BATs is likely to become more important in the years to come, as the laboratory investigation of individuals with excessive bleeding improves, and the use of BATs will allow comparison between patient groups and treatment centers. The fact that the ISTH-BAT documents recurrent minor bleeding episodes is important in the assessment of individuals with suspected inherited platelet function disorders.

Further work in this area should include international collaborations, such as the ISTH-BAT repository 17, to allow further validation of the ISTH-BAT and to obtain data on its performance in other hemorrhagic disorders.

## Addendum

G. Lowe and M. Lordkipanidzé: led on research governance for GAPP, recruited patients to the study, applied the ISTH-BAT, performed lumiaggregometry, analyzed and interpreted the results, and wrote the manuscript. S. P. Watson: chairs the GAPP program, interpreted the results, and edited the manuscript.
